# Effects of Subcutaneous Administration of Glucocorticoids by Pellets on a Mouse Model of Ligature-Induced Periodontal Disease

**DOI:** 10.3390/jcm14228251

**Published:** 2025-11-20

**Authors:** Rintaro Kato, Takuma Sato, Shunsuke Kako, Masako Tabuchi, Yuki Aoki, Kai Kataoka, Sho Okuni, Yoshihiko Sugita, Hatsuhiko Maeda, Ken Miyazawa

**Affiliations:** 1 Department of Orthodontics, School of Dentistry, Aichi Gakuin University, Nagoya 464-8651, Japan; gantzrin@icloud.com (R.K.);; 2 Department of Pediatric Dentistry, School of Dentistry, Aichi Gakuin University, Nagoya 464-8651, Japan; 3 Department of Oral Pathology, School of Dentistry, Aichi Gakuin University, Nagoya 464-8650, Japan

**Keywords:** glucocorticoids, periodontitis, osteoblasts, osteoclasts

## Abstract

**Background/Objective:** Glucocorticoids (GC) have anti-inflammatory effects, but long-term use can suppress bone formation and cause osteoporosis. The impact of inflammatory environments, such as periodontitis, on alveolar bone metabolism remains insufficiently understood. **Methods:** We used wild-type (C57BL/6J, *n* = 47) mice to compare glucocorticoid (GC) effects with and without sustained-release GC pellets. Mice were divided into GC-administered (2 weeks: *n* = 8; 4 weeks: *n* = 8; 8 weeks: *n* = 7) and non-GC-administered groups (2 weeks: *n* = 8; 4 weeks: *n* = 8; 8 weeks: *n* = 8). A ligature wire was placed around the left first molar of all mice to induce periodontal disease, while the right first molar served as a control. Femur and alveolar bone changes were assessed at 2, 4, and 8 weeks using μCT, HE staining, tartrate-resistant acid phosphatase (TRAP) staining, and immunohistochemistry (TNF-α). Anonymized evaluators performed histological analyses, and statistical analyses. One-way ANOVA with the Tukey post hoc test and *t* tests. **Results:** GC administration significantly reduced femoral bone mass at 2, 4, and 8 weeks. In mice without ligature, GC administration did not significantly affect alveolar bone mass or osteoblast number at 2 or 4 weeks, but a reduction was noted at 8 weeks post-treatment. No significant differences in osteoclast numbers or TNF-α levels were observed after GC administration. In a periodontal disease mouse model, GC administration led to greater bone loss, fewer osteoblasts, and increased osteoclasts and TNF-α levels. **Conclusions:** GC use in periodontal disease risks abnormal bone metabolism and progressive alveolar bone resorption.

## 1. Introduction

In recent years, the age range of patients undergoing orthodontic treatment has broadened, increasing the opportunities to provide orthodontic care to patients with compromised periodontal tissues, systemic diseases, and inflammatory conditions [1,2,3]. Although orthodontic treatment can help inhibit periodontal disease progression by dispersing occlusal forces and improving cleanability, orthodontic forces during treatment can exert mechanical stress on the periodontal ligament and alveolar bone, potentially causing localized inflammation and changes in bone metabolism [4]. In particular, applying orthodontic forces in the presence of inflammation has been reported to increase the load on periodontal tissues, potentially leading to unexpected bone resorption and tooth mobility [5].

Periodontal disease is a chronic inflammatory disease caused by bacteria in dental plaque and is one of the primary causes of tooth loss in adults. The progression of periodontal disease involves not only bacterial infection but also significant contributions from host immune responses and inflammatory reactions [6]. Specifically, the activation of inflammatory cytokines and immune cells promotes alveolar bone resorption and contributes to tissue destruction [7].

Glucocorticoids (GC) are steroid hormones secreted by the adrenal cortex that exert anti-inflammatory and immunosuppressive effects. Therefore, they are used to treat various inflammatory diseases, including autoimmune and allergic disorders. However, long-term use may cause osteoporosis, and its effects on bone metabolism have been reported [8].

Regarding the effects of glucocorticoids (GCs) on periodontal tissues and alveolar bone in rats and mice, de Almeida et al. reported that long-term and continuous administration of dexamethasone (a GC) increased alveolar bone resorption and exacerbated periodontal disease in a mouse model of periodontal disease, while also inducing spontaneous alveolar bone loss in otherwise healthy periodontal tissues [9]. Similarly, Bouvard et al. demonstrated that prolonged GC administration in mice reduced alveolar bone mass [10]. Furthermore, clinical studies have shown that GC treatment is associated with greater clinical attachment loss, higher probing depth, and lower bone density in the periodontium [11]. Several studies have reported that GCs promote alveolar bone resorption. In contrast, Baumeister et al. found that long-term plasma cortisol levels had no significant effect on the risk of developing periodontal disease [12]. Therefore, the effects of GC on periodontal diseases remain largely unknown. Although GC administration may suppress inflammation in the periodontal tissues owing to its anti-inflammatory effects, it may also promote alveolar bone destruction by accelerating bone resorption. Therefore, clarifying how GC affects the progression and treatment of periodontal disease is critical for understanding periodontal disease, developing treatment methods, and determining orthodontic treatment approaches.

This study aimed to investigate the effects of GC administration on alveolar bone in mice with and without periodontal disease.

## 2. Materials and Methods

### 2.1. Animals

Eight-week-old male WT mice (C57BL/6J) (total *n* = 47) were used as experimental animals in this study. The mice were purchased from Japan Crear Co., Ltd. (Tokyo, Japan), and housed in the Animal Laboratory of the School of Dentistry, Aichi Gakuin University. The housing environment maintained a room temperature of 22 ± 2 °C, humidity of 50 ± 10%, and a constant 12-h light-dark cycle. Tap water was used as drinking water, and CE-2 type solid feed (Nippon Crear Co., Ltd., Tokyo, Japan) was provided ad libitum. Animal management and research were conducted in accordance with the Animal Experiment Guidelines approved by the Aichi Gakuin University School of Dentistry Animal Experiment Committee (approval no. AGUD518, which was approved on 6 December 2023). Throughout the experimental period, all mice were monitored for general health, respiration, appearance, coat condition, and activity. Humane euthanasia was planned if the body weight decreased by more than 30%.

Mice were randomly divided into a GC-administered group (2 weeks: *n* = 8; 4 weeks: *n* = 8; 8 weeks: *n* = 7) and a non-GC-administered group (2 weeks: *n* = 8; 4 weeks: *n* = 8; 8 weeks: *n* = 8). A ligature wire was placed around the left first molar of all mice to induce a periodontal disease model, while the right first molar was left unligated and served as the control side.

This study had a randomized, single-anonymized, controlled design. The animals’ ears were numbered from 1 to 47. Data were organized and analyzed using Microsoft Excel (Microsoft Corp., Redmond, WA, USA). A blinded staff member not involved in the study performed simple randomization (1:1 allocation ratio) using a computer-generated number table to the groups.

### 2.2. Experimental Periodontal Disease Model Mice

**Figure 1:**
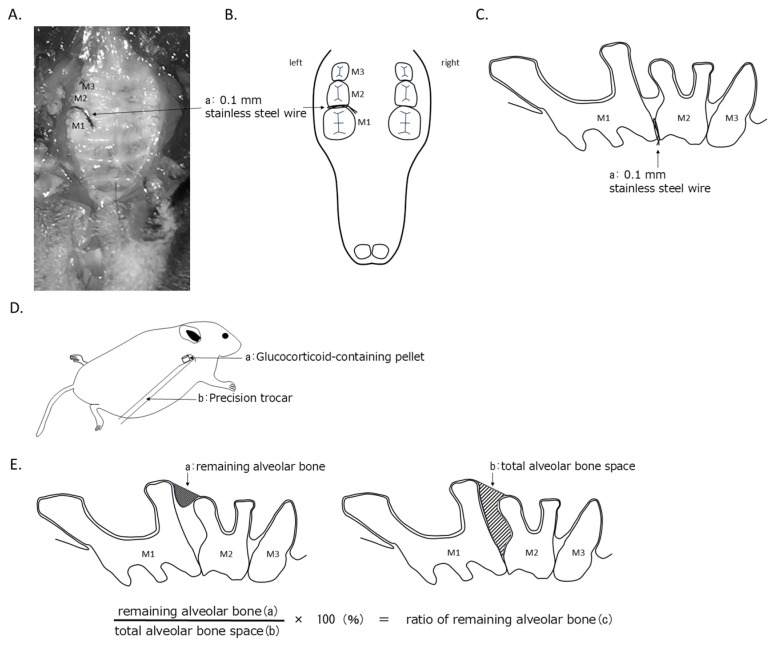
Schematic Diagram of Experimental Method. (**A**) Wire ligated at the contact point between the maxillary first molar and maxillary second molar (M1: maxillary first molar, M2: maxillary second molar, M3: maxillary third molar, a: 0.1 mm stainless steel wire). (**B**) Ligature site viewed from the occlusal surface (M1: maxillary first molar, M2: maxillary second molar, M3: maxillary third molar, a: 0.1 mm stainless steel wire). (**C**) Wire ligation site viewed from the buccal side (M1: maxillary first molar, M2: maxillary second molar, M3: maxillary third molar, a: 0.1 mm stainless steel wire). (**D**) Glucocorticoid-containing pellet implantation method (a: Glucocorticoid-containing pellet, b: Precision trocar). (**E**) Measurement site for alveolar bone residual volume between maxillary first and second molars (a: Remaining alveolar bone, b: Total alveolar bone space, c: Ratio of remaining alveolar bone).

The mice were locally anesthetized with a triple-drug combination of medetomidine hydrochloride (Meiji Seika Pharma, Tokyo, Japan), midazolam (Astellas Pharma, Tokyo, Japan), and butorphanol tartrate (Meiji Seika Pharma, Tokyo, Japan). Following the methods of Mizuno et al. [13] and Kimura et al. [14], food impaction was induced by ligating a 0.1 mm diameter stainless steel ligature wire (The Nilaco Co., Tokyo, Japan) around the contact point between the left maxillary first and second molars to create an experimental mouse model of periodontal disease (Figure 1A–C).

### 2.3. GC Administration

For the GC mouse model, sustained-release GC-containing pellets (5.0 mg/kg/day) manufactured by Innovative Research of America (Sarasota, Florida, USA) were used. Eight-week-old mice (*n* = 24) received subcutaneous pellet administration with a small incision using a pellet implantation device (PRECISION TROCHAR) (Figure 1D). The GC dosage was based on the study by Zhu et al. [15]. These pellets utilize a matrix-driven delivery system that releases a constant amount of hormone for up to 60 days post-implantation. The matrix is composed of cholesterol, cellulose, lactose, phosphates, and stearic acid, within which the GC is incorporated [16]. GC is released at a constant rate through both matrix erosion and diffusion [17]. Previous studies using GC pellets have reported that they are sufficient to induce bone loss within 28 days and cause body weight reduction within the first 7 days after implantation without affecting food intake [17,18,19]. In addition, Jia et al. reported that serum cortisol level was not changed at the 1.4 mg/kg/day GC dose level but increased at the 5.6 mg/kg/day dose level [20]. The placebo pellets contained the same matrix components as the GC pellets but lacked hormones. Subcutaneous administration was performed simultaneously with ligature wire ligation. The control group (*n* = 24) received placebo pellets administered subcutaneously using the same method. In this experiment, the pellet was implanted only once at the beginning, and no replacement was performed during the 8-week observation period.

### 2.4. μCT Imaging

Maxillary bones and left femurs were harvested 2 (*n* = 16), 4 (*n* = 16), and 8 weeks (*n* = 15) after ligature wire placement and imaged using μCT (Rigaku, Tokyo, Japan). The imaging conditions were as follows: tube voltage, 90 kV; tube current, 88 μA; scan time, 2 min; pixel size, 20 × 20 × 20 μm. Alveolar bone residual volume measurements and analyses were performed using TRI/3D-BON software (version R.12.00.03.9-H-64; Ratoc System Engineering, Osaka, Japan). To measure the residual volume of the alveolar bone, referring to the methods of Park et al. [21] and Takeguchi et al. [22], as shown in Figure 1E, the residual alveolar bone volume within each root apex was measured from the contact point between the maxillary first and second molars.

The ratio of this volume to the total volume in the same region was defined as the residual alveolar bone ratio (Figure 1E). After μCT scanning, analysis was performed on 20 consecutive slices, each 20 μm thick, as described by Kimura et al. [14]. The femoral bone volume was measured and analyzed using the same software used for alveolar bone volume measurement. After μCT scanning, analysis was performed on 100 consecutive slices, each 50 μm thick, as described by Yoshizako et al. [23]. The cortical bone contours were traced semi-automatically and excluded. The bone volume/total tissue volume (BV/TV) ratio was calculated and defined as the femoral bone remaining rate.

### 2.5. Histopathological Observation

The collected maxillary and femoral bones were fixed in 10% neutral-buffered formalin. Next, demineralization was performed in 10% EDTA (pH 7.2) for approximately 4 weeks at 4 °C. Following standard procedures, the specimens were paraffin-embedded, and 5 μm serial tissue sections were prepared in the apocentric direction. The region for histological observation was selected such that all the roots of the molar region were visible. Hematoxylin and eosin (HE) staining was used to measure osteoblast numbers. The number of osteoblasts (Ob.N/BS [mm]) on the alveolar ridge surface in the alveolar septum between the first and second molars was counted. Tartrate-resistant acid phosphatase (TRAP) staining was performed using a TRAP staining kit (FUJIFILM Wako Pure Chemical Corporation, Osaka, Japan). The number of osteoclasts (Oc. N/BS [mm]) on the surface of the alveolar ridge in the alveolar septum between the first and second molars was measured. Furthermore, to assess inflammatory responses, immunostaining for tumor necrosis factor-alpha (TNF-α) was performed. Immunohistochemical staining was performed using Histofine Simple Stain Mouse MAX-PO (Nichirei Bioscience Inc., Tokyo, Japan) and Histofine Simple Stain DAB Solution (Nichirei Bioscience Inc., Tokyo, Japan). The primary antibody was anti-TNF alpha (ab34674, TNF-α: 1/100, Abcam Inc., Waltham, MA, USA), and the VECTASTAIN Elite ABC Mouse IgG Kit (Vector Laboratories, Burlingame, CA, USA) was used as the secondary antibody. Immunohistochemical staining was performed as described by Rogers et al. [24]. The percentage of stained area on the alveolar ridge surface of the alveolar septum between the first and second molars was used to classify the samples into four groups: 0–20%, 21–40%, 41–60%, and ≥61%, with scores of 1–4, respectively. Two independent evaluators scored the stained images. (Kimura et al. [14], Kataoka et al. [25])

### 2.6. Statistical Analysis

Experimental data were expressed as mean ± standard deviation. Statistical significance was assessed using Student’s *t* test for the femoral bone and a one-way analysis of variance (ANOVA) with the Tukey multiple-comparison test for the other experiments.

All statistical analyses were performed using IBM SPSS Statistics Version 30 (IBM, Armonk, NY, USA). Statistical significance was set at *p* < 0.05.

Two independent evaluators repeated the histologic and IHC endpoints 4 weeks later to evaluate measurement errors. A *t*-test was used to compare the corresponding samples, and no statistically significant systematic errors were found for any of the variables (*p* > 0.05).

## 3. Results

### 3.1. Comparison of Femoral Bone Remaining Rate in μCT Images (Figure 2A,B)

**Figure 2:**
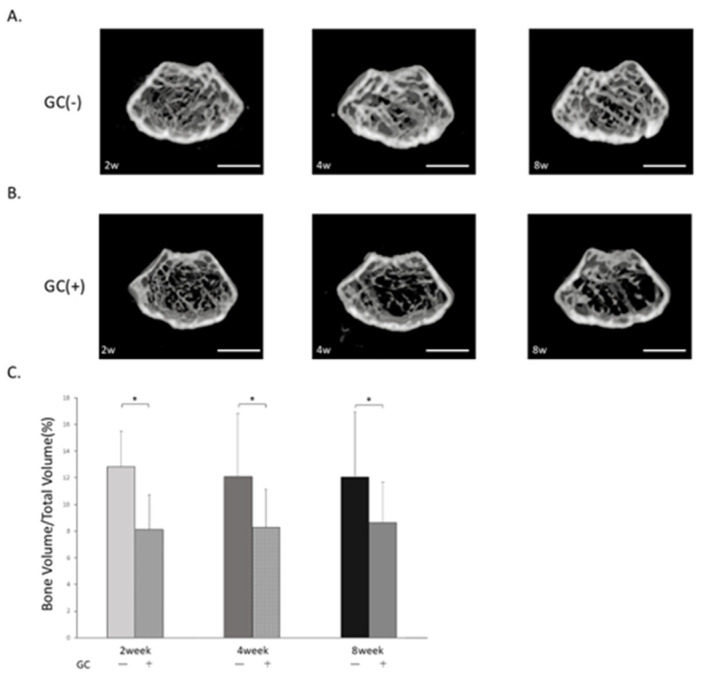
Femoral bone remaining rate by μCT imaging. (**A**) μCT image without GC administration. (**B**) μCT image with GC administration. (**C**) Femoral bone remaining rate (%) *: *p* < 0.05. Scale bar: 1000 μm.

The group administered GC (2 weeks: 8.1 ± (2.6)%, 4 weeks: 8.3 ± (2.9)%, 8 weeeks: 8.6 ± (3.1)%) showed a significantly reduced femoral bone remaining rate at 2, 4, and 8 weeks compared to the group not administered GC (2 weeks: 12.8 ± (2.7)%, 4 weeks: 12.1 ± (4.8)%, 8 weeks: 12.1 ± (4.9)%) (Figure 2C).

### 3.2. Comparison of Alveolar Bone Remaining Rate in μCT Images (Figure 3)

#### 3.2.1. Comparison Based on Presence/Absence of Ligation

**Figure 3:**
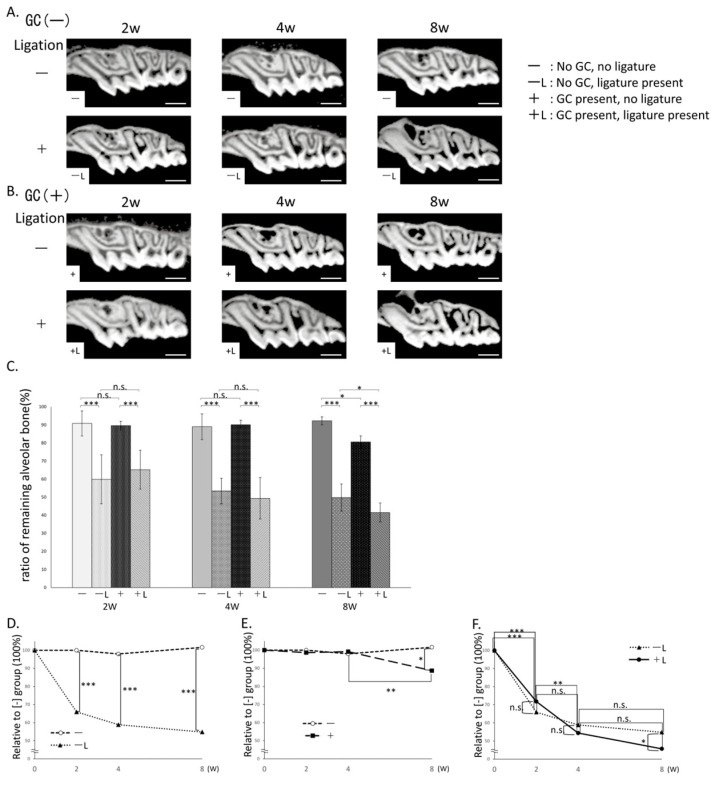
Alveolar bone remaining rate by μCT imaging. (**A**) μCT images of no GC administration without ligature wire (−) (top) and no GC administration with ligature wire (−L) (bottom). (**B**) μCT images of GC administration with no ligature wire (+) (top) and GC administration with ligature wire (+L) (bottom). (**C**) Alveolar bone remaining rate (%) between first and second molars. n.s.: not significant, *: *p* < 0.05, ***: *p* < 0.001. (**D**) Comparison based on ligature presence (Percentage relative to 100% alveolar bone remaining rate in no-ligature wire group without GC; No GC administration without ligature wire (−) (◯), no GC administration with ligature wire (−L) (▲)) ***: *p* < 0.001. (**E**) Comparison with or without GC administration (Percentage relative to 100% alveolar bone remaining rate in no-ligature wire group without GC; No GC administration without ligature wire (−) (◯), GC administration without ligature wire (+) (■) *: *p* < 0.05, **: *p* < 0.01. (**F**) Time-dependent changes with or without GC administration in the ligature wire group (Percentage relative to 100% alveolar bone remaining rate in no-ligature wire group without GC; GC administration with ligature wire (+L) (●), no GC administration with ligature wire (−L) (▲)) n.s.: not significant, *: *p* < 0.05, **: *p* < 0.01, ***: *p* < 0.001. Scale bar: 1000 μm.

The group with ligature applied but without GC administration (hereafter, the −L group) showed a significantly reduced alveolar bone remaining rate at 2, 4, and 8 weeks compared to the group without ligature (hereinafter, the—group) (Figure 3C,D).

Similarly, the group with ligature placement and GC administration (+L group) showed a significantly reduced alveolar bone remaining rate at 2, 4, and 8 weeks compared to the group without ligature placement (+group) (Figure 3C).

#### 3.2.2. Comparison with and Without GC Administration

No significant difference in alveolar bone remaining rate was observed between the +(2 weeks: 89.6 ± (2.5)%; 4 weeks: 90.1 ± (2.5)%) and −groups (2 weeks: 90.9 ± (6.9)%; 4 weeks: 89.0 ± (7.1)%) at 2 and 4 weeks. However, at 8 weeks, the +group (80.6 ± (3.4)%) showed a significantly lower remaining rate than the −group (92.3 ± (2.2)%) (Figure 3C,E). Similarly, no significant differences were observed between the +L (2 weeks: 65.3 ± (10.8)%; 4 weeks: 49.4 ± (11.5)%) and −L groups (2 weeks: 59.8 ± (13.6)%; 4 weeks: 53.5 ± (7.1)%) at 2 and 4 weeks. However, at 8 weeks, the +L group (41.5 ± (5.3)%) showed a significantly lower rate than the −L group (49.8 ± (7.5)%) (Figure 3F).

Furthermore, comparison of the −L and +L groups over time revealed a significant decrease in the alveolar bone remaining rate from 0 to 2 weeks in both groups. From weeks 2 to 4, although no significant differences were observed in the −L group, a gradual decrease was noted. In contrast, the +L group exhibited a significant decrease. Furthermore, from weeks 4 to 8, although no significant difference was observed between the −L and +L groups, both groups showed a gradual decrease. Consequently, at 8 weeks, the +L group showed a significant decrease compared to the −L group (Figure 3F).

### 3.3. Measurement of Osteoblast Counts (Figure 4)

#### 3.3.1. Comparison Based on Presence/Absence of Ligation

**Figure 4:**
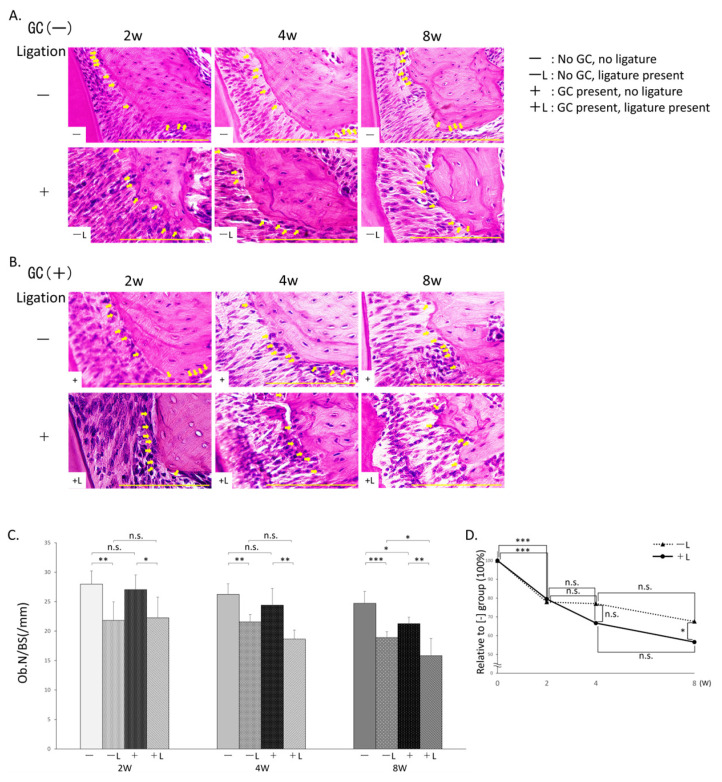
Osteoblast count by hematoxylin and eosin (HE) staining. (**A**) HE-stained histological images of no GC administration without ligature wire (−) (top) and no GC administration with ligature wire (−L) (bottom). (**B**) HE-stained histological images of GC administration without ligature wire (+) (top) and GC administration with ligature wire (+L) (bottom). (**C**) Comparison of osteoblast numbers (%). n.s.: not significant, *: *p* < 0.05, **: *p* < 0.01, ***: *p* < 0.001. (**D**) Time-dependent changes with or without GC administration in the presence of ligature wire (Percentage relative to the osteoblast count in the no-ligature wire group without GC administration set to 100: GC administration with ligature wire (+L) (●), no GC administration with ligature wire (−L) (▲)) n.s.: not significant, *: *p* < 0.05, ***: *p* < 0.001. Scale bar: 200 μm. Magnification: 400×. The arrows indicate osteoblasts.

The −L group (2 weeks: 21.8 ± (3.2)/mm, 4 weeks: 21.5 ± (1.3)/mm, 8 weeks: 18.9 ± (1.0)/mm) showed significantly reduced osteoblast counts compared to the −group (2 weeks: 28.0 ± (2.2)/mm, 4 weeks: 26.2 ± (1.8)/mm, 8 weeks: 24.7 ± (2.0)/mm) at 2, 4, and 8 weeks. Similarly, the +L group (2 weeks: 22.2 ± (3.5)/mm, 4 weeks: 18.7 ± (1.5)/mm, 8 weeks: 15.8 ± (2.9)/mm) showed significantly reduced osteoblast counts compared to the +group (2 weeks: 27.0 ± (2.5)/mm, 4 weeks: 24.4 ± (2.8)/mm, 8 weeks: 21.3 ± (1.1)/mm) at 2, 4, and 8 weeks (Figure 4C).

#### 3.3.2. Comparison with or Without GC Administration

The +group showed no significant difference in osteoblast number compared with the −group at 2 and 4 weeks, but did show a significant decrease at 8 weeks. Conversely, the +L group showed no significant difference in osteoblast numbers compared to the −L group at 2 and 4 weeks, but a significant decrease was observed at 8 weeks (Figure 4C).

Comparing the −L and +L groups over time, both groups showed a significant decrease in osteoblast numbers from week 0 to week 2. From weeks 2 to 4, the −L group showed almost no significant difference, whereas the +L group showed a decreasing trend, although no significant difference was observed in the latter. At week 4, the +L group showed a decreasing trend compared to the −L group, although this difference was not statistically significant. Subsequently, from weeks 4 to 8, both the −L and +L groups showed a decreasing trend at the same rate. Consequently, at week 8, the +L group had significantly fewer cells than the −L group (Figure 4D).

### 3.4. Measurement of Osteoclast Numbers (Figure 5)

#### 3.4.1. Comparison Based on Presence/Absence of Ligation

**Figure 5:**
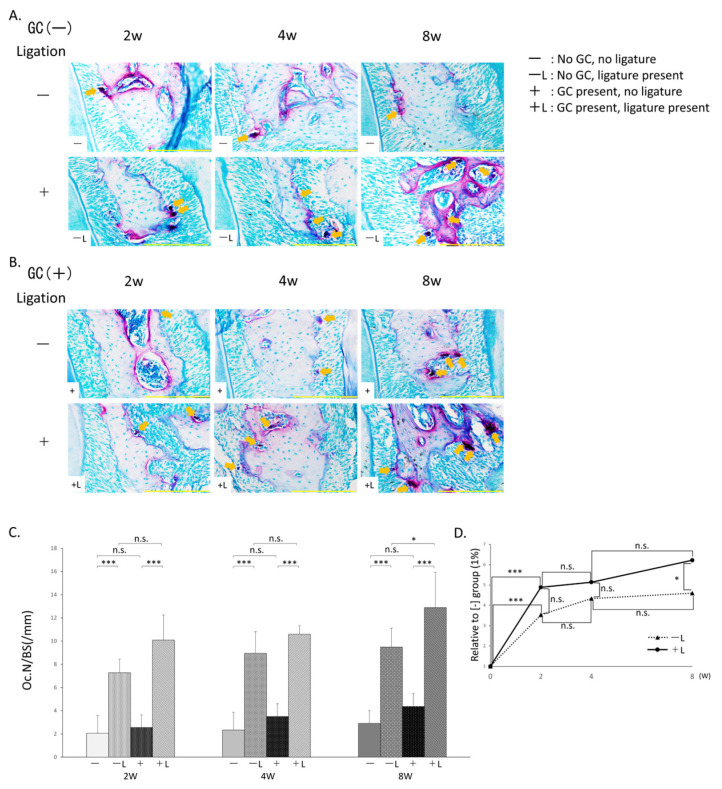
Osteoclast count by Tartrate-resistant acid phosphatase (TRAP) staining. (**A**) TRAP-stained tissue images of no GC administration without ligature wire (−) (top) and no GC administration with ligature wire (−L) (bottom). (**B**) TRAP-stained tissue images of GC administration without ligature wire (+) (top) and GC administration with ligature wire (+L) (bottom). (**C**) Comparison of osteoclast numbers (%) n.s.: not significant, *: *p* < 0.05, ***: *p* < 0.001. (**D**) Time-dependent changes with or without GC administration in the presence of ligature wire (Percentage relative to the osteoclast count in the no-ligature wire group without GC administration set to 1: GC administration with ligature wire (+L) (●), no GC administration with ligature wire (−L) (▲)) n.s.: not significant, *: *p* < 0.05, ***: *p* < 0.001. Scale bar: 200 μm. Magnification: 400×. The arrows indicate osteoclast.

The −L group (2 weeks: 7.3 ± (1.2)/mm, 4 weeks: 8.9 ± (1.9)/mm, 8 weeks: 9.5 ± (1.6)/mm) showed a significant increase in osteoclast numbers compared to the −group (2 weeks: 2.1 ± (1.5)/mm, 4 weeks: 2.3 ± (1.5)/mm, 8 weeks: 2.9 ± (1.1)/mm) at 2, 4, and 8 weeks. Similarly, the +L group (2 weeks: 10.1 ± (2.2)/mm, 4 weeks: 10.6 ± (0.7)/mm, 8 weeks: 12.8 ± (2.6)/mm) showed a significant increase in osteoclast numbers compared to the +group (2 weeks: 2.6 ± (1.1)/mm, 4 weeks: 3.5 ± (1.1)/mm, 8 weeks: 4.4 ± (1.1)/mm) at 2, 4, and 8 weeks (Figure 5C).

#### 3.4.2. Comparison with or Without GC Administration

The +group showed no significant difference in osteoclast number compared to the −group at 2, 4, and 8 weeks, but an increasing trend in osteoclast numbers was observed. In contrast, the +L group showed no significant difference in osteoclast numbers compared to the −L group at 2 and 4 weeks, but a significant increase was observed at 8 weeks (Figure 5C).

Comparing the −L and +L groups over time, both groups showed a significant increase in osteoclast numbers from 0 to 2 weeks. Although no significant difference was observed at week 2, the +L group had approximately 1.4 times as many osteoclasts as the −L group. Furthermore, in weeks 2 to 4 and 4 to 8, both the −L and +L groups showed an increasing trend at the same rate. Consequently, at 8 weeks, the +L group showed a significantly higher number of osteoclasts than the −L group (Figure 5D).

### 3.5. Comparison of Staining Scores for TNF-α Using Immunohistochemistry (Figure 6A,B)

#### 3.5.1. Comparison Based on Presence/Absence of Ligation

**Figure 6:**
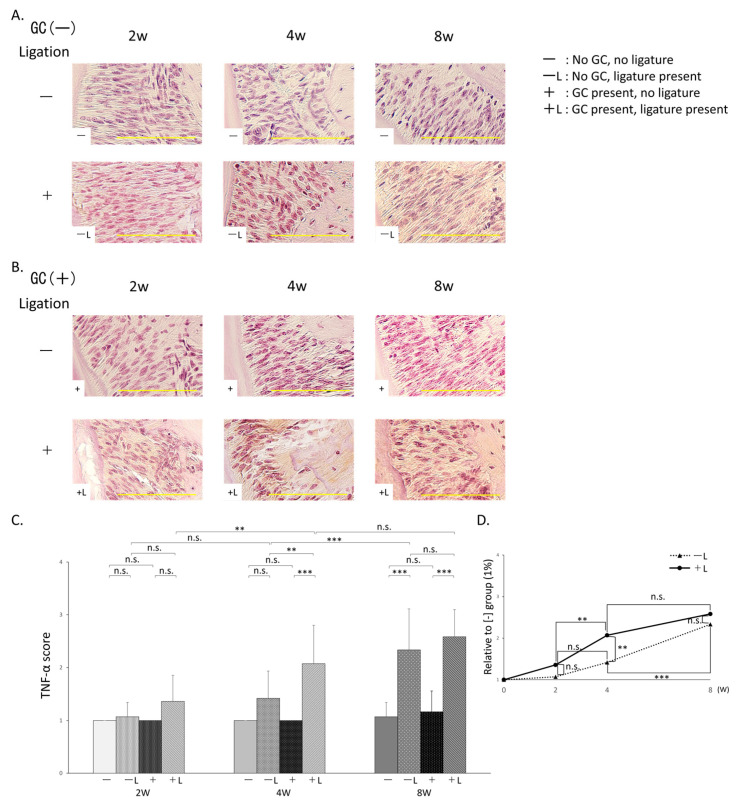
TNF-α Immunohistochemical Staining. (**A**) TNF-α immunostaining tissue images of no GC administration without ligature wire (−) (top) and no GC administration with ligature wire (−L) (bottom). (**B**) TNF-α immunostaining tissue images of GC administration without ligature wire (+) (top) and GC administration with ligature wire (+L) (bottom). (**C**) TNF-α staining scores: n.s.: not significant, **: *p* < 0.01, ***: *p* < 0.001. (**D**) Time-dependent changes with or without GC administration in the presence of ligature wire (Percentage relative to the staining score of the no-ligature wire group without GC administration set to 1: GC administration with ligature wire (+L) (●), no GC administration with ligature wire (−L) (▲)) n.s.: not significant, **: *p* < 0.01, ***: *p* < 0.001. Scale bar: 100 μm. Magnification: 400×.

The −L group (2 weeks: 1.1 ± (0.3), 4 weeks: 1.4 ± (0.5), 8 weeks: 2.3 ± (0.8)) showed no significant difference in staining scores compared to the −group (2 weeks: 1.0 ± (0), 4 weeks: 1.0 ± (0), 8 weeks: 1.1 ± (0.3)) at 2 and 4 weeks but showed a significant increase at 8 weeks. The +L group (2 weeks: 1.4 ± (0.5), 4 weeks: 2.1 ± (0.7), 8 weeks: 2.6 ± (0.5)) showed no significant difference in staining scores compared to the +group (2 weeks: 1.0 ± (0), 4 weeks: 1.0 ± (0), 8 weeks: 1.2 ± (0.4)) at 2 weeks but showed significant increases at both 4 and 8 weeks (Figure 6C).

#### 3.5.2. Comparison with or Without GC Administration

The +group showed no significant difference in staining scores compared to the −group at 2, 4, and 8 weeks post-treatment. In contrast, the +L group showed no significant difference in staining scores compared with the −L group at 2 and 8 weeks, but did show a significant increase at 4 weeks (Figure 6C). Furthermore, when comparing the −L and +L groups over time, no significant differences in the staining scores were observed between 0 and 2 weeks in either group. From 2 to 4 weeks, no significant change was observed in the −L group, whereas a significant increase was observed in the +L group. From 4 to 8 weeks, the −L group showed a significant increase, whereas no significant difference was observed in the +L group (Figure 6C,D).

## 4. Discussion

### 4.1. Differences in Whole-Body Bone Responses to GC Administration

Various types of bones are present in the body. Vertebrae, such as the lumbar vertebrae, are formed via endochondral ossification, whereas the parietal bone is formed via membranous ossification [26]. Furthermore, in long bones such as the femur, the epiphysis forms by endochondral ossification, whereas the diaphysis primarily increases in diameter by membranous ossification [27]. Thus, bone quality varies site-specifically. Additionally, it has been reported that bone metabolic activity differs, with the parietal bone exhibiting a slower metabolic turnover rate than other tissues [28]. This study focused on the femur and alveolar bones. In the femur, a significant reduction in bone mass was observed starting 2 weeks after GC administration and continuing through week 8. This is attributed to the femur being classified as a long bone, with its shaft composed of thick cortical bone and its epiphysis containing extensive cancellous bone, which has been reported to be sensitive to systemic regulation of bone metabolism [29]. Furthermore, Yao et al. reported that GC administration in mice reduced trabecular bone mass early, attributed to simultaneous activation of osteoclasts and inhibition of osteoblasts from the initial stage [30]. Furthermore, Kim et al. revealed that GC suppresses bone formation via osteoclasts [31], a mechanism thought to underlie the early bone mass reduction observed in the femur.

In contrast, in alveolar bone treated with GC without inducing periodontal disease, no significant differences were observed at 2 and 4 weeks post-administration; however, a reduction in alveolar bone volume and osteoblast number was noted at 8 weeks. Regarding the differences observed at 2 and 4 weeks compared to the femur, the alveolar bone, like the femoral epiphysis, is largely cancellous and consistently receives mechanical stimuli, such as occlusal forces and mastication, via the periodontal ligament. Mechanical stimulation is considered a contributing factor to this phenomenon. Mechanical stimulation promotes bone formation [32]. Therefore, in the alveolar bone, even after GC administration, bone loss was less likely to occur at 2 and 4 weeks, and no significant difference was observed. However, when GC was administered for 8 weeks, the previously reported bone-forming inhibitory effect [10,33,34,35] likely occurred, leading to a significant reduction in alveolar bone volume.

### 4.2. Relationship Between GC Administration and Periodontal Disease

When GC was administered after periodontal disease induction, no significant difference was observed at 2 weeks; however, the +L group had a higher alveolar bone volume than the −L group. Subsequently, in the +L group, a marked decrease was observed between 2 and 4 weeks. At 4 weeks, the +L group tended to have a lower volume than the −L group, indicating a reversal in alveolar bone volume. By week 8, the +L group showed a significant reduction compared to the −L group. There was little change in the osteoblast numbers in the −L group from weeks 2 to 4, whereas the +L group showed a decreasing trend. By week 8, the +L group had significantly fewer osteoblasts than the −L group.

Regarding osteoclast count, no significant difference was observed at week 2, but the +L group had approximately 1.4 times as many osteoclasts as the −L group. Subsequently, at week 8, the +L group showed a significant increase compared to the −L group. Thus, the reversal in alveolar bone volume observed between weeks 2 and 4 was considered to result from a decrease in the osteoblast count and an increase in the osteoclast count in the alveolar bone. Furthermore, TNF-α, an indicator of inflammation, significantly increased in the +L group from week 2 to week 4. At week 4, the +L group exhibited significantly higher levels than those of the −L group. This suggests that GC administration exacerbates inflammation at an early stage in a mouse model of periodontal disease.

Previous studies have shown that periodontal disease is caused by bacteria present in dental plaque, which induce the production of inflammatory cytokines, such as TNF-α, IL-1β, and IL-6, thereby forming a cytokine cascade [36,37]. In the early stages, macrophages produce TNF-α and IL-1β, which, via IL-6, increase RANKL expression, thereby promoting osteoclast differentiation [38]. In this study, we focused on TNF-α, which is expressed in the early phase of the inflammatory response. In addition, Kanzaki et al. reported that osteoclasts become predominant under inflammatory conditions, accelerating bone resorption [39]. Furthermore, although molecular assays (gene/protein) were not performed in this experiment, GC has been reported to suppress bone formation by inhibiting RUNX2 and Wnt signaling [33] and inducing apoptosis in osteoblasts and osteoclasts [19]. Thus, factors promoting bone resorption due to the inflammatory environment [38,39,40] and the bone formation-suppressing effects of GC [10,33,34,35] overlap and synergistically accelerate bone loss. The alveolar bone is constantly subjected to occlusal forces and masticatory stimuli through the periodontal ligament. While some reports indicate that mechanical stimulation promotes bone formation [32], Wang et al. reported that mechanical loading under inflammatory conditions exacerbates bone destruction [4]. Therefore, in this experiment, the inflammatory environment under GC administration was shown to carry the risk of accelerating bone loss early and rapidly by (1) promoting osteoclast formation through inflammatory cytokines, (2) suppressing osteoblasts and promoting osteoclasts via GC administration, and (3) enhancing bone resorption due to mechanical stimuli, such as occlusal forces.

However, this study had limitations in its research findings. First, this was a basic study using mice. Second, the GC administration method used a fixed dose via sustained-release pellets, which may differ from oral or injectable clinical administration methods. Therefore, its effects on bone metabolism may differ depending on the dose, duration of administration, and pharmacokinetic profile. Third, the observation periods were set at 2, 4, and 8 weeks; the long-term effects beyond this period are unknown. Although GCs exhibit anti-inflammatory effects in the acute phase, suppression of bone formation becomes prominent in the chronic phase [41], necessitating detailed follow-up.

The present results revealed that, in a mouse model of periodontal disease, GC administration accelerated alveolar bone loss, starting approximately 2 weeks after administration, due to the overlapping effects of bone formation suppression and inflammation-induced bone resorption. Although further research is needed, clinically, when orthodontic treatment is performed in the presence of periodontal disease, GC administration exacerbates alveolar bone loss. Therefore, preventing the onset and progression of periodontal disease is crucial, requiring thorough plaque control and, if necessary, regular evaluation of the periodontal tissues to ensure safe treatment with a favorable prognosis.

## 5. Conclusions

This study examined the effects of subcutaneous GC administration on alveolar bone and bone metabolism in a mouse model of periodontal disease. The results showed that in the femur, systemic GC effects led to progressive bone mass reduction starting at 2 weeks, with ongoing bone resorption throughout the 8 weeks. In contrast, in the alveolar bone, bone mass was maintained at 2 and 4 weeks in the nonperiodontal disease model mice. However, at 8 weeks, significant inhibition of bone formation was observed, confirming the reduction in bone mass. Furthermore, in a periodontal disease model, increases in inflammatory cytokines and in GC bone formation and resorption acted synergistically. This resulted in a more advanced reduction in alveolar bone mass from 2 to 4 weeks compared to the periodontal disease model without GC treatment. These results indicate that the use of GCs in periodontal disease carries the risk of inducing abnormalities in bone metabolism and promoting alveolar bone resorption, leading to progressive alveolar bone loss.

## Data Availability

All data supporting the findings of this study are contained within this article and its figures. No additional datasets were generated or analyzed in the current study.

## Abbreviations

The following abbreviations are used in this manuscript:

GC	glucocorticoid
TNF-α	Tumor necrosis factor-alpha
μCT	micro–computed tomography
HE	hematoxylin and eosin
TRAP	tartrate-resistant acid phosphatase
BV/TV	bone volume/total volume
WT	wild type
ANOVA	analysis of variance
SD	standard deviation
RUNX2	Runt-related transcription factor 2
Wnt	Wingless/integrated signaling pathway
